# Comprehensive network medicine-based drug repositioning via integration of therapeutic efficacy and side effects

**DOI:** 10.1038/s41540-022-00221-0

**Published:** 2022-04-20

**Authors:** Paola Paci, Giulia Fiscon, Federica Conte, Rui-Sheng Wang, Diane E. Handy, Lorenzo Farina, Joseph Loscalzo

**Affiliations:** 1 Department of Computer, Control and Management Engineering, Sapienza University of Rome, Rome, Italy; 2 Institute for Systems Analysis and Computer Science “Antonio Ruberti”, National Research Council, Rome, Italy; 3 Department of Medicine, Brigham and Women’s Hospital, Harvard Medical School, Boston, MA 02115 USA

**Keywords:** Virtual drug screening, Cardiology, Drug discovery

## Abstract

Despite advances in modern medicine that led to improvements in cardiovascular outcomes, cardiovascular disease (CVD) remains the leading cause of mortality and morbidity globally. Thus, there is an urgent need for new approaches to improve CVD drug treatments. As the development time and cost of drug discovery to clinical application are excessive, alternate strategies for drug development are warranted. Among these are included computational approaches based on omics data for drug repositioning, which have attracted increasing attention. In this work, we developed an adjusted similarity measure implemented by the algorithm SAveRUNNER to reposition drugs for cardiovascular diseases while, at the same time, considering the side effects of drug candidates. We analyzed nine cardiovascular disorders and two side effects. We formulated both disease disorders and side effects as network modules in the human interactome, and considered those drug candidates that are proximal to disease modules but far from side-effects modules as ideal. Our method provides a list of drug candidates for cardiovascular diseases that are unlikely to produce common, adverse side-effects. This approach incorporating side effects is applicable to other diseases, as well.

## Introduction

Cardiovascular disease (CVD) is a group of disorders of the heart and blood vessels, including coronary and peripheral arterial disease, cerebrovascular disease, congenital heart disease, heart failure, hypertension, and arrhythmias. CVD is consistently ranked as the leading cause of mortality and morbidity in the United States and globally. According to the World Health Organization (WHO), 17.9 million people die each year from CVDs, an estimated 32% of all deaths worldwide. Despite remarkable progress in addressing CVD, the decline in population mortality rates is slowing and cardiovascular drug innovation is lagging. There is, therefore, an urgent need for new approaches to improve CVD drug treatments^1,2^.

New drug development from compound identification to application in the clinic is a lengthy and costly process. Drug repositioning (or repurposing), a process of using an existing drug for a new indication, has numerous advantages over conventional drug discovery^3^. High-throughput screening approaches^4,5^ and computational drug repositioning approaches^6,7^ are currently used to repurpose approved drugs. In particular, unbiased systems pharmacology methods using omics data to reposition drugs computationally has attracted increasing attention^8,9^. Notwithstanding the success of these approaches, a crucial issue in the repurposing of approved drugs is the possible occurrence of unexpected side effects. While many computational methods have been proposed for drug repositioning, few of them incorporate an analysis of potential side effects^10–13^.

Prevailing opinion holds that repurposing approved drugs is a comparatively safe process owing to the known side-effect profile of those compounds. Yet, this tenet fails to take into consideration the potential for new or unrecognized side effects that are unveiled in the context of the drug’s use for a new disease (drug-by-disease interaction). Moreover, often a drug’s side effects are not observed during clinical trials but emerge only after approval when a much larger number of patients begin to use it^14,15^.

In this work, we present a new approach to the identification of repurposed drug candidates that incorporates protein interaction network-based analysis of therapeutic efficacy and side effects. By classifying potentially repurposable drugs in terms of efficacy and adverse effects, we can identify the most promising candidates based on optimal benefit and safety. As a proof-of-concept, we apply this strategy to repurposing drugs for cardiovascular diseases and do so while utilizing a similar network-based approach to two side effects that often appear among cardiovascular medications (i.e., electrocardiographic QT interval prolongation and drug-induced asthma).

Our approach is based on the notion that, much like diseases themselves, drug side effects are exerted through discrete subnetworks or modules within the interactome (which we demonstrate here for the serious side-effect of electrocardiographic QT interval prolongation, a harbinger of sudden cardiac death; and for drug-induced asthma). The rationale underlying our analysis is analogous to the proximity hypothesis for drug repurposing for disease—the closer a drug target is to a disease module in the interactome, the greater the likelihood that the drug can be repurposed for the disease^8,16^. Similarly for drug-induced side effects, we propose that the closer a drug target is to a side-effect module, the greater the likelihood that the drug will induce the side-effect. From a primary disease module perspective for a drug target that is proximal both to a disease module and a side-effect module, the closer the drug target is to a side-effect module, the greater the likelihood that the drug will not only produce the side-effect, but may also attenuate the benefit of drug action. This effect is particularly likely to occur if a side-effect is manifest in the same general disease cluster as the disease for which the drug is being repurposed (e.g., a cardiovascular side-effect for a drug repurposed for a cardiovascular disease). From a primary side-effect module perspective for a drug that is proximal both to a side-effect module and a disease module, the closer a drug target is to a disease module, the greater the likelihood that the drug will be effective in treating the disease, but may also attenuate the side-effect (competitive pathway effects within proximate modules in the interactome).

This analysis can, thereby, provide insight into a previously unrecognized risk (or benefit) of drug repurposing, viz., that a side-effect may become apparent that was not previously recognized (positive synergy) or a known side-effect may be attenuated (negative synergy)—both because the drug target is close to both the (repurposed) disease module and to the side-effect module. This situation can highlight a unique risk (or benefit) that reflects (positive or negative) synergism between the side-effect and the disease.

Our method produced a list of drug candidates for cardiovascular diseases that are unlikely to produce well-known adverse effects. This approach predicting repurposable drugs and incorporating potential side effects in the process is applicable to other diseases, as well.

## Results

### Drug-disease network

In the present study, we applied the SAveRUNNER algorithm^16,17^ to identify repurposable drug candidates for cardiovascular diseases. In particular, we studied a panel of nine cardiovascular diseases or (a) disease-equivalent (i.e., arrhythmia, cardiomyopathies, cardiac arrest, coronary artery disease, coronary heart disease, angina pectoris, myocardial infarction, cerebral arterial disease, and diabetes mellitus) and two possible side-effects (i.e., long QT syndrome and drug-induced asthma). As input, SAveRUNNER requires a list of drug targets and a list of disease genes to evaluate the extent to which a given drug can be eventually repositioned to treat a disease. Here, the disease-associated genes were downloaded from Phenopedia^18^ and DisGeNet^19^, whereas drug-target associations were obtained from DrugBank^20^ (cf. Methods).

**Fig. 1:**
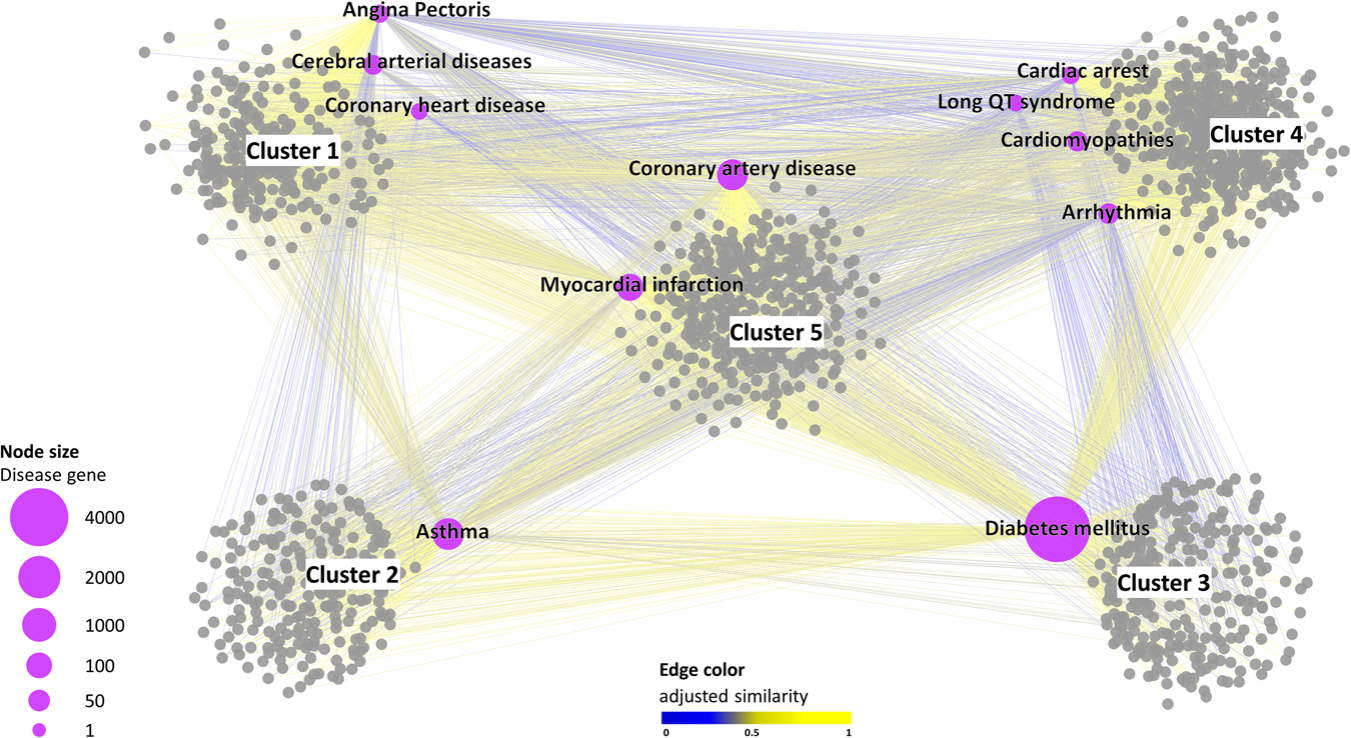
Drug-disease network. This diagram shows the high-confidence predicted drug-disease associations connecting 11 diseases (labeled purple circles) with the 1541 FDA-approved drugs (gray circles). The node size scales with the number of disease-associated genes. The edge color denotes the adjusted similarity between drug targets and disease genes in the human interactome, increasing from blue (less similar) to yellow (more similar). For the clusters’ identification on the drug-disease network, SAveRUNNER exploits a cluster detection algorithm based on greedy optimization of the network modularity^47^. The five clusters identified by SAveRUNNER are highlighted with the corresponding labels.

The rationale behind SAveRUNNER lies in the hypothesis that, for a drug to be effective for a specific disease, its associated targets (drug module) and the disease-specific-associated genes (disease module) should be nearby in the human interactome^21^. To quantify the vicinity between drug and disease modules, SAveRUNNER implements a novel network similarity measure, called the adjusted similarity measure, rewarding drugs and diseases that fall in the same neighborhood, and assesses the statistical significance by applying a degree-preserving randomization procedure^17^. As output, SAveRUNNER releases a weighted bipartite drug-disease network, where a link between a drug and a disease occurs if the corresponding drug targets and disease genes are closer in the human interactome than expected by chance (i.e., *z*-score normalized values of the network proximity ≤ −1.65), and the weight of their interaction corresponds to the adjusted similarity measure (cf. Methods)^16,17^. In this study, the drug-disease network was composed of 1552 nodes (i.e., 11 diseases and 1541 drugs) connected by 6436 links (Supplementary Table 1 and Fig. 1).

For elucidating drugs/diseases relatedness in terms of network similarity, SAveRUNNER performs a cluster analysis on the drug-disease network to detect groups of drugs and diseases in such a way that members in the same group (cluster) are more similar to each other than to those in other groups (clusters). This analysis highlighted five main clusters: cluster one, which includes angina pectoris, cerebral arterial diseases, and coronary heart disease; cluster two, which includes asthma; cluster three, which includes diabetes mellitus; cluster four, which includes cardiomyopathies, cardiac arrest, arrhythmia, and the long QT syndrome; and cluster five, which includes coronary artery disease and myocardial infarction.

Using both a greedy partitioning clustering algorithm (Fig. 1) and a complete linkage hierarchical clustering algorithm (Supplementary Fig. 1), we observed that the long QT syndrome fell in the cluster of cardiomyopathies, while asthma was in a different cluster well-separated from cardiomyopathies. This finding has face validity as drug-induced asthma, unlike the long QT syndrome, is a side-effect not directly linked to cardiovascular biology or diseases.

### Identification of network modules

As well-established by network medicine principles^22–24^, disease-associated genes have unique, quantifiable characteristics that distinguish them from other genes. This observation can be translated into the verification that disease-associated genes do not map randomly in the interactome but, rather, agglomerate in locally dense and topologically well-defined regions of this network (denoted *disease modules*), whose nodes show an increased tendency to interact with each other more frequently than expected by chance. In order to verify this property, we mapped the list of genes associated with the 11 analyzed diseases and side effects to the human interactome, and verified that they constitute statistically significant disease modules (Table 1).

**Table 1:** Module search results.

Disease	LCC size		LCC interactions		Total interactions	
	Observation	*p*-value	Observation	*p*-value	Observation	*p*-value
Angina pectoris	103	2.78E-26	150	2.78E-38	151	2.56E-46
Arrhythmia	179	1.98E-24	276	1.23E-43	293	7.40E-68
Cardiomyopathies	215	2.66E-18	515	3.37E-47	525	1.13E-53
Coronary artery disease	796	3.63E-17	2446	2.47E-123	2469	9.21E-128
Coronary heart disease	4	0.009	3	0.01	3	0.026
Diabetes mellitus	3293	0.006	26029	3.75E-209	26035	1.83E-210
Cardiac arrest	61	1.01E-22	88	1.14E-42	92	4.16E-50
Myocardial infarction	593	5.54E-19	1821	8.62E-92	1832	2.75E-93
Cerebral arterial diseases	172	1.07E-08	371	2.11E-26	377	6.18E-28
Long QT syndrome	16	1.71E-90	16	4.63E-93	17	2.86E-53
Asthma	961	2.66E-10	4447	1.12E-87	4457	1.63E-89

The size of the largest connected component (LCC); the number of interactions in the LCC; and the total number of interactions are reported for each disease, along with the corresponding *p*-values resulting from a degree-preserving randomization procedure (cf. Methods).

### Side-effects estimation

In order to estimate potential side-effects of the repurposable drugs predicted by SAveRUNNER for a certain disease, we defined a new pipeline that relies on the hypothesis that exploring the network-based relationship between drug targets, disease genes, and side-effect-associated genes in the human interactome would help clarify the mechanism-of-action of effective drugs while minimizing adverse effects. The basic premise of this exercise is that just as in the network neighborhood of disease modules there are candidate drug targets^25^, in the network neighborhood of drug targets there are possible side-effect modules.

**Fig. 2:**
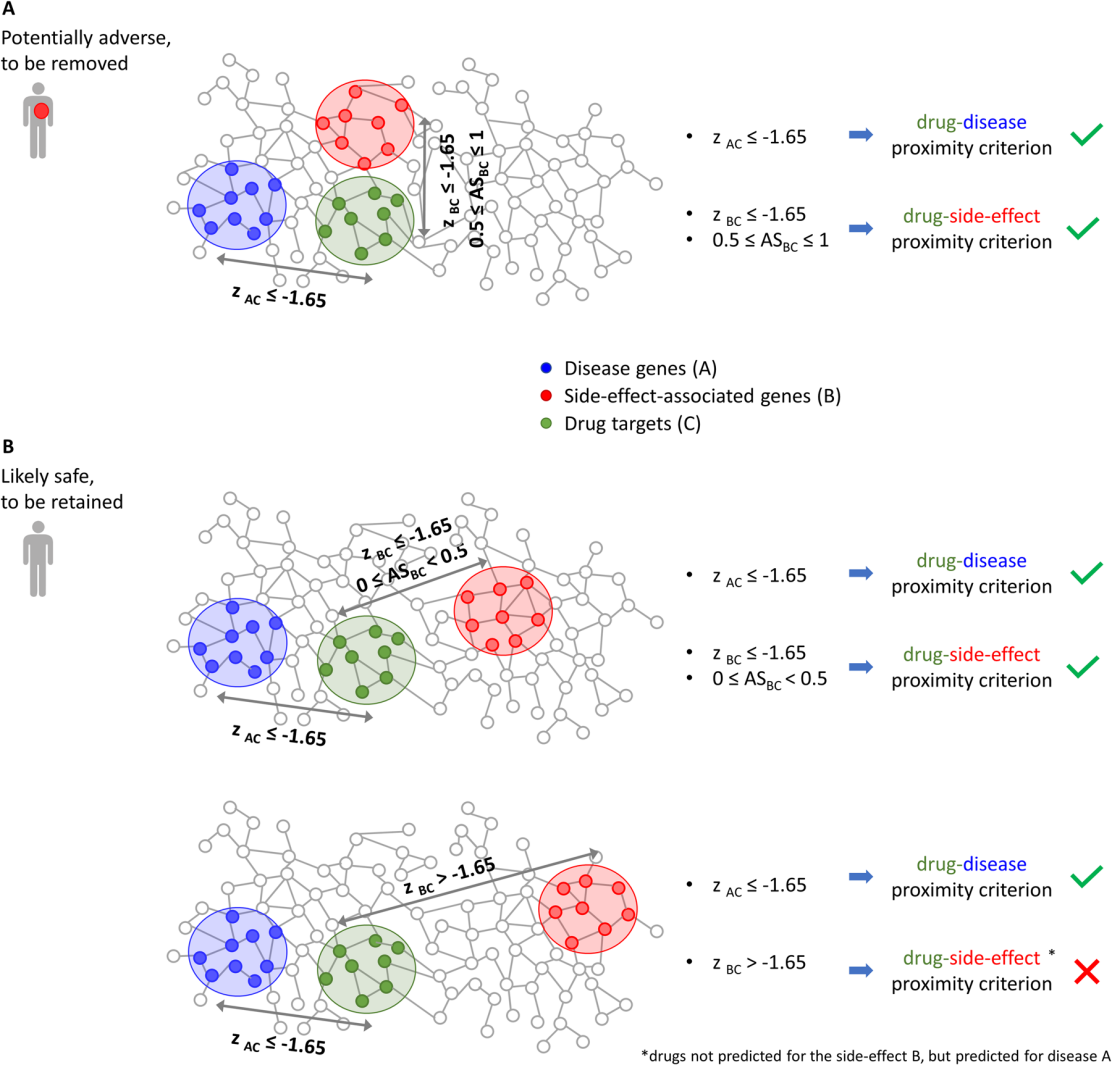
Study design. The figure depicts the topological relationships among the drug module (green), the disease module (blue), and the side-effect module (red) for a drug to be removed from (panel **A**) or retained for (panel **B**) consideration. The *z*_AC_ and *z*_BC_ denote the *z*-score normalized network proximity between the targets of the drug (C) and the genes associated with the disease (**A**) and with the side-effect (**B**), respectively. AS_BC_ is the adjusted similarity between the drug targets (C) and the side-effect-associated genes (**B**). In the bottom row, the AS_BC_ value is not reported for drugs with *z*_BC_ > −1.65 since they are removed independent of their AS_BC_ values.

We derived the side-effect information from the SIDER database^26^ and/or from the published literature^27,28^ (cf. Methods). By doing so, we identified drugs that may likely be harmful and, thus, should be removed from consideration for repurposing for the disease of interest (Fig. 2). The pipeline consists of two steps that, for disease A and side-effect B, are the following:

1. **Drug-disease proximity criterion**. Removal of the drugs whose targets have a *z*-score normalized value for network proximity > −1.65 with respect to disease A.
2. **Drug-side-effect proximity criterion**. Removal of drugs predicted to be repurposable for disease A (i.e., whose targets have a *z*-score normalized value for network proximity ≤ −1.65 with disease A) and whose targets have an adjusted similarity ≥ 0.5 for side-effect B (Fig. 2A).

**Fig. 3:**
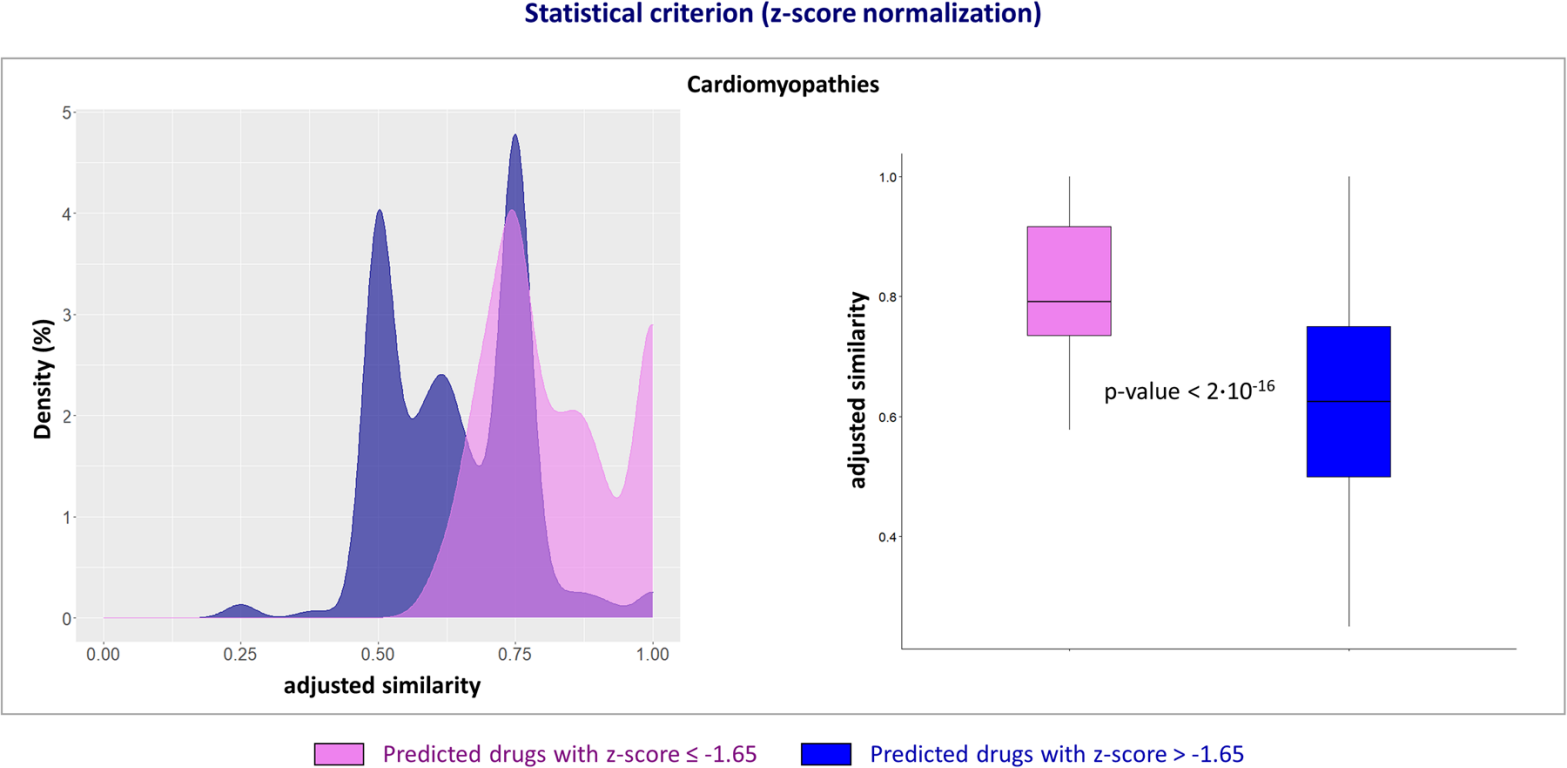
Drug-disease proximity criterion. Kernel density estimate plots (left) and box plots (right) for the adjusted similarity values of the statistically significant drugs (pink plot, corresponding to a *z*-score ≤ −1.65) and those not statistically significant (blue plot, corresponding to a *z*-score > −1.65) predicted by SAveRUNNER as repurposable for cardiomyopathies. *t*-test was used to compare the two distributions, and statistical significance was found (*p*-value < 0.05). Box-plot elements are so defined: center line, median; box limits, upper and lower quartiles; points, outliers.

**Fig. 4:**
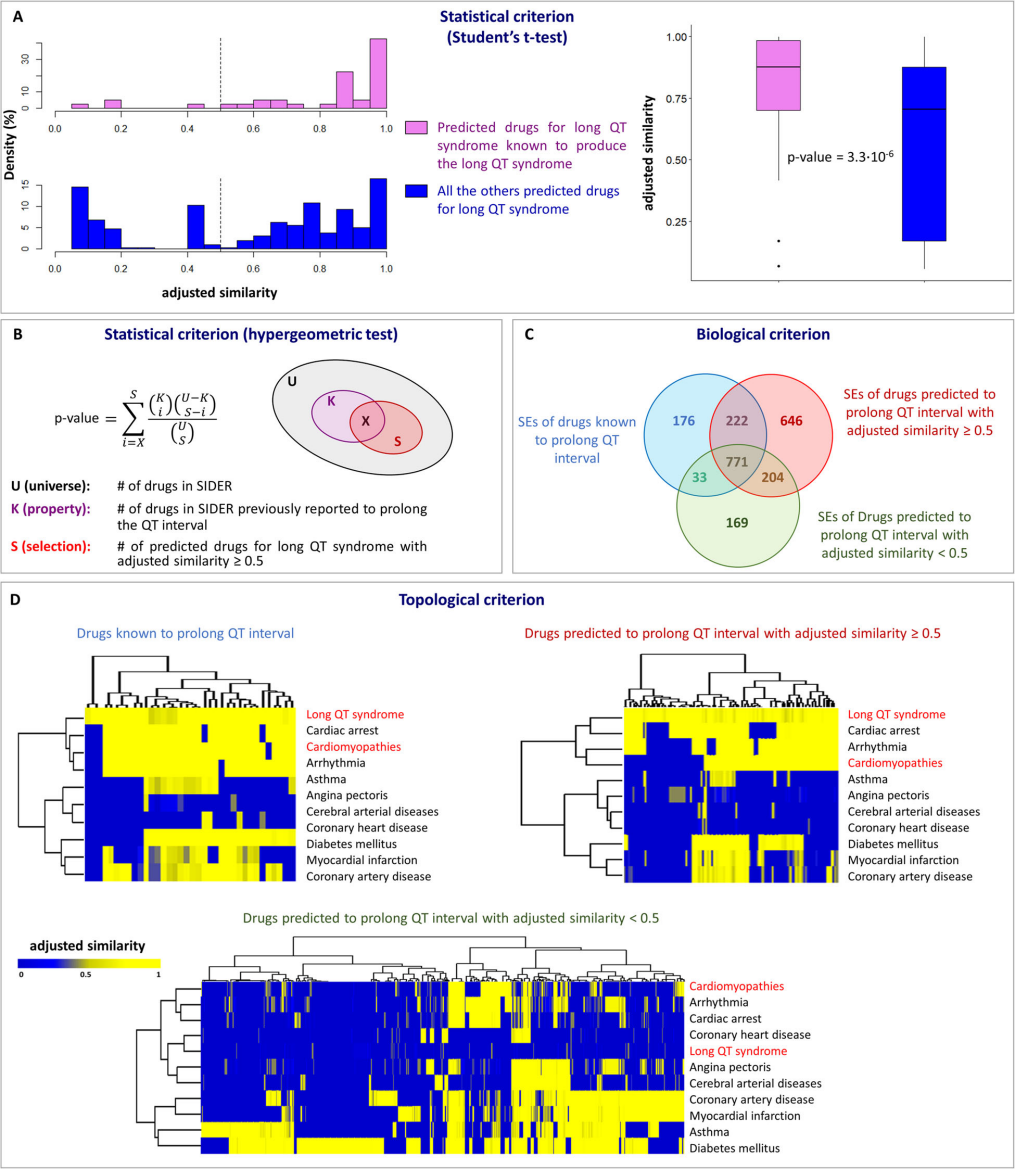
Drug-side-effect proximity criterion. **A** Statistical criterion (Student’s *t*-test): histogram (left) and box plots (right) of the adjusted similarity values of the drugs predicted by SAveRUNNER as repurposable regarding their predicted effects on long QT syndrome, of the drugs known to induce long QT syndrome (pink bar), and of all the other predicted drugs (blue bar). *t*-test was used to compare the two distributions. Box-plot elements are so defined: center line, median; box limits, upper and lower quartiles; points, outliers. **B** Statistical criterion (hypergeometric test): sketch of the ensembles considered for the hypergeometric test calculation. **C** Biological criterion: Venn diagram among the side-effects (SEs) of the drugs known to prolong QT interval (blue ensemble), of the drugs predicted by SAveRUNNER to prolong QT interval with adjusted similarity ≥0.5 (red ensemble) and with adjusted similarity <0.5 (green ensemble). **D** Topological criterion: dendrogram and heatmap of the drug-disease network predicted by SAveRUNNER including drugs known to prolong QT interval (top left), drugs predicted to prolong QT interval with adjusted similarity ≥0.5 (top right), and drugs predicted to prolong QT interval with adjusted similarity <0.5 (bottom).

The definition of the drug-disease proximity criterion stems from the observation that the predicted drugs for the diseases analyzed in this study with a *z*-score ≤ −1.65 showed higher adjusted similarity values than those with a *z*-score > 1.65, and, thus, the corresponding drug targets’ modules and disease genes’ modules are more proximal to each other in the human interactome (Fig. 3 left and Supplementary Fig. 2). The observed difference between the two adjusted similarity distributions was tested to be statistically significant (Student’s *t*-test *p*-value < 0.05, Fig. 3, right). The definition of the drug-side-effect proximity criterion derives from the following statistical, biological, and topological analyses, using the long QT syndrome as a serious adverse side-effect to be avoided:

i. **Statistical criterion (Student’s** ***t*-test)**—Among the predicted drugs for the long QT syndrome with a *z*-score ≤ −1.65, those that were known to prolong the QT interval from the SIDER database^26^ or literature studies^27,28^ showed a higher adjusted similarity value (Fig. 4A left) with respect to all of the other predicted drugs. Thus, the corresponding drug-targets’ modules and the side-effect-associated genes’ module are more proximal to each other in the human interactome. The observed difference between the two adjusted similarity distributions was found to be statistically significant (Student’s *t*-test *p*-value < 0.05, Fig. 4A, right).
ii. **Statistical criterion (hypergeometric test)**—Drugs predicted to prolong the QT interval with an adjusted similarity value ≥0.5 were statistically enriched in drugs previously demonstrated to prolong the QT interval (*p*-value = 8.57 × 10^−7^, Fig. 4B).
iii. **Biological criterion**—Drugs known to prolong the QT interval shared a higher percentage of other side-effects with drugs predicted to prolong the QT interval with adjusted similarity value ≥0.5 (222 of 993 other side-effects, corresponding to 22%) compared with those with adjusted similarity value <0.5 (33 of 804 other side-effects, corresponding to 4%) (Fig. 4C).
iv. **Topological criterion**—Rendering the drug-disease network obtained by using SAveRUNNER as a drug-disease-adjusted similarity matrix, the hierarchical clustering structure along the rows (i.e., diseases) is preserved when considering drugs known to induce long QT syndrome and predicted drugs with adjusted similarity value ≥0.5 for long QT syndrome (Fig. 4D top), still remaining different from those obtained considering predicted drugs with adjusted similarity value <0.5 for long QT syndrome (Fig. 4D bottom).

In total, our methodology predicts 363 drugs for long QT syndrome. Among them, 238 have an adjusted similarity value ≥0.5 (Supplementary Table 2), suggesting that they are potentially adverse and including 36 drugs known to induce long QT syndrome (true positives). By contrast, 125 of the 363 predicted drugs have an adjusted similarity value <0.5 (Supplementary Table 3), suggesting that they are likely safe and including 4 drugs known to induce long QT syndrome (false negatives). To assess the statistical significance of these results, we performed the receiver operating characteristic (ROC) curve analysis and we found over 70% accuracy (AUC = 0.72) for identifying drugs known to induce long QT syndrome (Supplementary Fig. 3A).

Thus, considering the drugs predicted by SAveRUNNER as repurposable for all of the analyzed cardiovascular diseases, we removed those with an adjusted similarity value ≥0.5 for the long QT syndrome arguing that it may likely prolong the QT interval as predicted by our algorithm (Supplementary Table 3). We obtained ~70% accuracy (AUC = 0.70) for identifying known drug-disease associations, as shown by the ROC curve analysis (Supplementary Fig. 3B).

Drug-induced asthma, unlike the long QT syndrome, is not directly linked to cardiovascular diseases (i.e., its side-effect module is distant from the cardiomyopathies module, as a model cardiovascular disease). On this basis, we observed that the statistical, biological, and topological criteria were still satisfied (Supplementary Fig. 4). In particular, those drugs predicted to induce asthma with adjusted similarity values ≥ 0.5 are enriched in drugs known to produce it (*p*-value = 6.58 × 10^−6^) and, thus, we removed them from the list of drugs to be repurposed for the diseases analyzed in this study (Supplementary Table 2 and Supplementary Table 4).

In total, our methodology predicts 704 drugs for asthma. Among them, 682 have an adjusted similarity value ≥0.5 (Supplementary Table 2), suggesting that they are potentially adverse and including 85 drugs known to induce asthma (true positives). By contrast, 22 of the 704 predicted drugs have an adjusted similarity value <0.5 (Supplementary Table 4), suggesting that they are likely safe, and including 5 drugs known to induce asthma (false negatives).

### Drugs’ mode of action

**Fig. 5:**
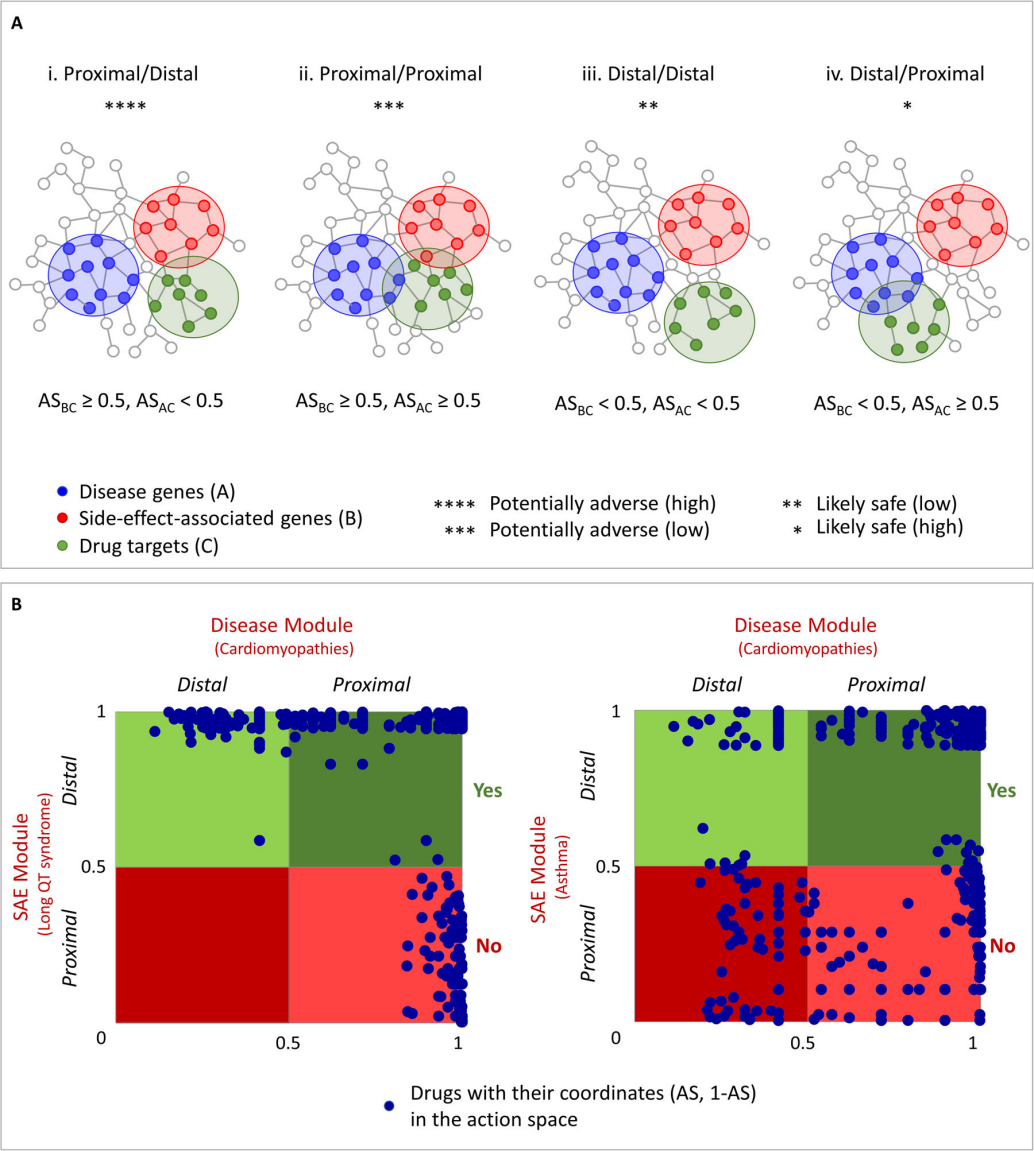
Drug actions as function of distance of drug target to disease module or to serious adverse effect (SAE) module. **A** Drug target distance analysis. Four possible modes of action of the drug based on the distance of the drug module (**C**) to the disease module (**A**) and the SAE module (**B**) are depicted according to the corresponding adjusted similarity value (AS). Stars indicate severe/beneficial effects of the different drug’s mode-of-action. **B** Drug-action map. Scatter plot of AS for the disease module (i.e., cardiomyopathies) on the *x*-axis, and (1-AS) for SAE module (i.e., [left] long QT syndrome, [right] drug-induced asthma) on the *y*-axis. The four possible modes of action are colored with shades of red and green according to their predicted adverse or beneficial effects, respectively.

According to our pipeline and from a network perspective, we found that all possible modes of action of a drug can be classified into four topologically distinct classes (Fig. 5A): the targets of the drug are in the near neighborhood of the side-effect module, but far from the disease module in the human interactome (i.e., proximal/distal); the targets of the drug are in the near neighborhood both of the side-effect module and the disease module (i.e., proximal/proximal); the targets of the drug are far from the side-effect module, but in the near neighborhood of the disease module (i.e., distal/proximal); and the targets of the drug are far from both the side-effect module and the disease module (i.e., distal/distal). The proximal/distal mode-of-action corresponds to a situation in which the adjusted similarity values between side-effect-associated genes and drug targets are ≥0.5, while those between disease-associated genes and drug targets are <0.5; the proximal/proximal (distal/distal) mode-of-action corresponds to a situation in which the adjusted similarity values between side-effect-associated genes and drug targets, as well as between disease-associated genes and drug targets, are ≥0.5 (<0.5); and the distal/proximal mode-of-action corresponds to a situation in which the adjusted similarity values between side-effect-associated genes and drug targets are <0.5, while those between disease-associated genes and drug targets are ≥0.5 (Supplementary Table 5). The adjusted similarity values of the drug targets with respect to the side-effect-associated genes are then plotted against those of the drug targets with respect to the disease-associated genes, creating a so-called *drug-action map*, colored according to the four modes of drug action (Fig. 5B).

Among the drugs removed from our pipeline owing to predicted side-effects (shades of red in Fig. 5B), the most adverse are those whose targets are distal to the disease module and, therefore, not likely to be beneficial for the disease (dark red in Fig. 5B); whereas those drugs whose targets are proximal to the disease module may be considered less adverse owing to potential concomitant therapeutic benefit (red in Fig. 5B). Nonetheless, a drug target near a side-effect module, whether or not it is close to a disease module, remains potentially adverse clinically when the side-effect is serious and viewed independent of the disease process. By contrast, predicted drugs that are likely safely repurposable for the disease are those whose targets are distal to the side-effect module (shades of green in Fig. 5B). Among them, the most beneficial are those whose targets are most proximal to the disease module (dark green in Fig. 5B); whereas those drugs whose targets are distal to the disease module have a lower chance of benefit, while preserving an absence of risk (green in Fig. 5B).

## Discussion

In this study, we emphasize the difference between beneficial drug effects and (serious) adverse effects in relation to drug repurposing as guided by network topology. In particular, we focused on nine cardiovascular diseases or disease-equivalents and on two possible side-effects (i.e., long QT syndrome, and drug-induced asthma), which could be induced by the repositioning of approved drugs. In order to predict repurposable drugs for the diseases of interest, we used SAveRUNNER, a recently developed network-based algorithm for drug repurposing that offers a list of candidate drugs by rewarding associations between drugs and diseases that are located in the same network neighborhood. SAveRUNNER builds a drug-disease network, where nodes are drugs and diseases; a link occurs if the corresponding drug targets and disease genes are closer in the human interactome than expected by chance, with their association weighted by the adjusted similarity measure (Fig. 1). In order to estimate possible adverse effects of the eligible drugs predicted by SAveRUNNER, here, we developed a novel pipeline that exploits the adjusted similarity values to quantify the interplay among drug targets, disease genes, and side-effect-associated genes in the human interactome.

The basic premise of this exercise is that just as a drug’s target proteins should be within or in the immediate vicinity of the corresponding disease module for it to be effectively repurposed for a specific disease^8,17^, for a side-effect to be induced by a specific drug, its associated genes should be within or in the immediate vicinity of the corresponding drug target module. In particular, by studying the adjusted similarity values of the drug targets with respect to the side-effect-associated genes in combination with those of the drug targets with respect to the disease-associated genes, four modes of beneficial/adverse drugs action were identified based on the distance of the drug module to the side-effect and disease module (Fig. 5A). This pipeline enables one to exclude as potentially adverse drugs that are proximal to the side-effect module in the human interactome and distal or proximal to the disease module, as illustrated in the drug-action map (Fig. 5B). That is, a proximity relationship between the side-effect module and drug target predicts an increased likelihood that the drug induces the side-effect and, thus, a distal relationship would be preferred. However, the complex drug/disease/side-effect interactions (not directly accounted for in the model) may mitigate this interpretation in some circumstances, *viz*., that a side-effect may become apparent that was not previously recognized (positive synergy) or a known side-effect may be attenuated (negative synergy), in both cases because the drug target is close to both the (repurposed) disease module and to the side-effect module. In this sense, the light red quadrant of the action diagram (Fig. 5B) corresponding to the proximal/proximal mode-of-action, can highlight a unique side-effect risk that reflects (positive or negative) synergism between the side-effect and the disease. For example, in the case of long QT syndrome and sudden cardiac death, it is well-known that pre-existing cardiomyopathy puts one at increased risk of sudden death from non-cardiac drug-induced QT interval prolongation^29^ (positive synergism), and network analysis provides a molecular mechanism by which to explain this synergistic adversity.

The predictions of our pipeline show a very different pattern for potentially repurposable drugs for cardiomyopathies vs. long QT syndrome (Fig. 5B, left) or asthma (Fig. 5B, right). In the case of long QT syndrome, we observed a lack of drugs populating the proximal/distal mode of action (i.e., dark red region in Fig. 5B, left), with the distribution of the adjusted similarity values with respect to cardiomyopathies is less sparse and densified at the top of the graph (i.e., light green region in Fig. 5B left). By contrast, for asthma, we observed a greater distribution in the adjusted similarity values of drugs that populated all the four regions, corresponding to all four possible modes of drug action (Fig. 5B, right). This pattern still holds even when considering another cardiovascular disease, such as arrhythmia (Supplementary Fig. 5 and Supplementary Table 6).

This observation reflects a fairly glaring difference in the intrinsic nature of the two considered side-effects. On the one hand, the long QT syndrome is a very precise phenotype, quantitatively measured on an electrocardiogram. In addition, the mechanisms by which the QT interval is prolonged are well integrated with other known mechanisms for other cardiovascular diseases, including cardiomyopathies. On the other hand, asthma is not a very quantitative or precise phenotype, characterized by greater variations in the complexity and diversity of its inducing causes and phenotypic manifestations or consequences.

This difference between phenotypes leads the long QT syndrome and drug-induced asthma to fall into two different clusters in the drug-disease network computed by SAveRUNNER, with the long QT syndrome located in the same cluster as cardiomyopathies (Fig. 1). This location is a direct consequence of the similarity in the distribution of the drug-disease associations between the long QT syndrome and cardiomyopathies, as distinct from that of asthma. Rewarding the former and penalizing the latter, SaveRUNNER paves the way for a different density distribution of the drug-action map (Fig. 5B).

**Fig. 6:**
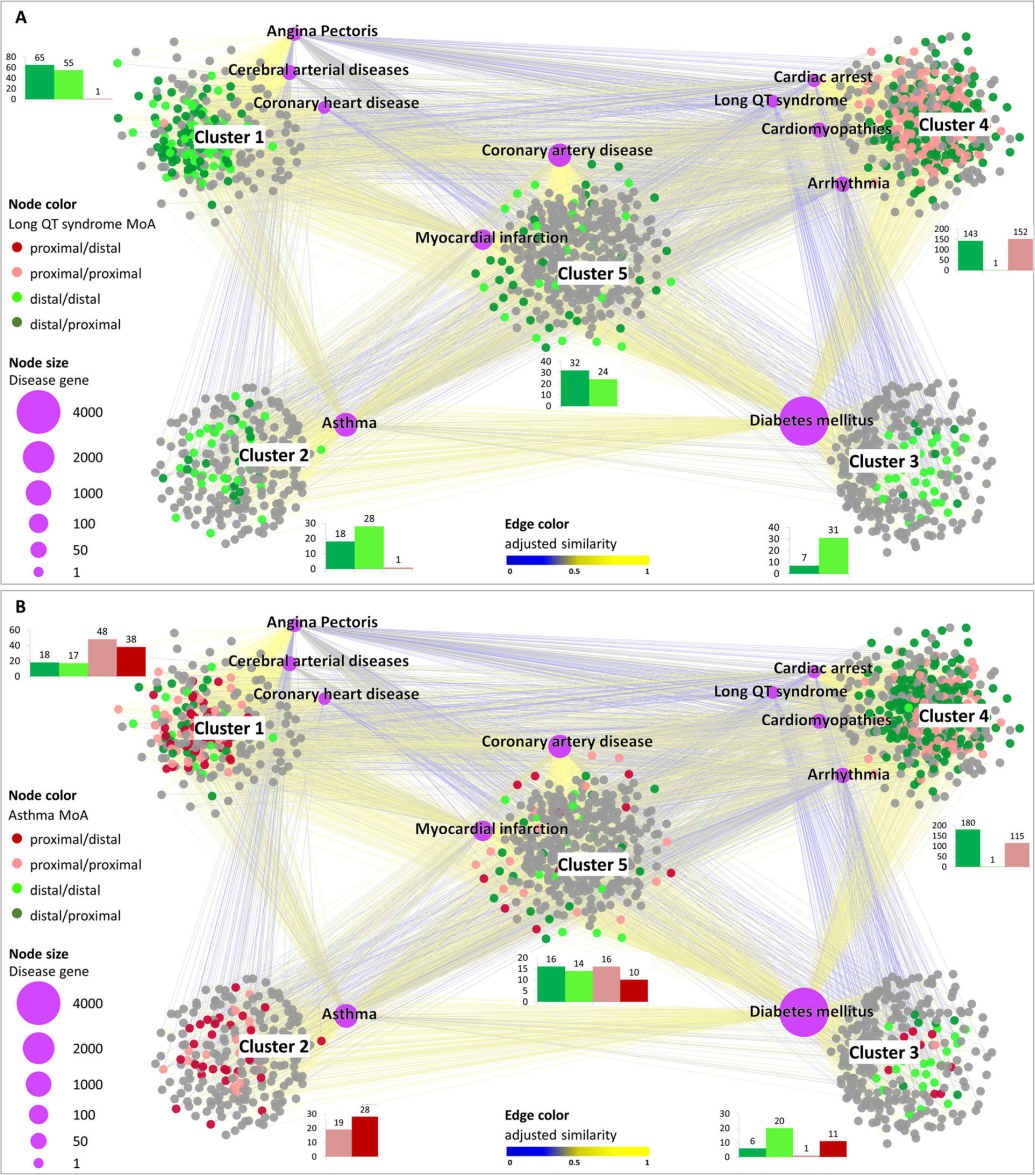
Drug-disease network. This illustration shows the high-confidence predicted drug-disease associations connecting 11 diseases (labeled purple circles) with the 1,541 FDA-approved drugs (gray circles), where drugs are colored according their mode-of-action (MoA) with respect to long QT syndrome (**A**) and asthma (**B**) SAEs. The node size scales with the number of disease-associated genes. The edge color denotes the adjusted similarity between drug targets and disease genes in the human interactome, increasing from blue (less similar) to yellow (more similar). For the clusters identification on the drug-disease network, SAveRUNNER exploits a cluster detection algorithm based on greedy optimization of the network modularity^47^. The five clusters identified by SAveRUNNER are highlighted with the corresponding labels. The bars of the histogram next to each network cluster show the count of the node types (i.e., distal/proximal, distal/distal, proximal/proximal, proximal/distal) within that cluster and are colored according to the MoA color legend.

When the drug-disease network is displayed with the four possible modes of drug action, we found for the long QT syndrome an absence of dark red nodes (proximal/distal drugs) since the targets of potentially adverse drugs that are in its nearest neighborhood cannot be far from the cardiomyopathies, their being in the same cluster (Fig. 6A). By contrast, in the case of the drug-induced asthma, we observed a conspicuous presence of dark red nodes uniformly distributed among all clusters of the network except for that containing the long QT syndrome and cardiomyopathies. This finding is interpreted to mean that the targets of potentially adverse drugs that are in the nearest neighborhood of the asthma module are far from the cardiomyopathies module, their being in a different cluster (Fig. 6B).

**Fig. 7:**
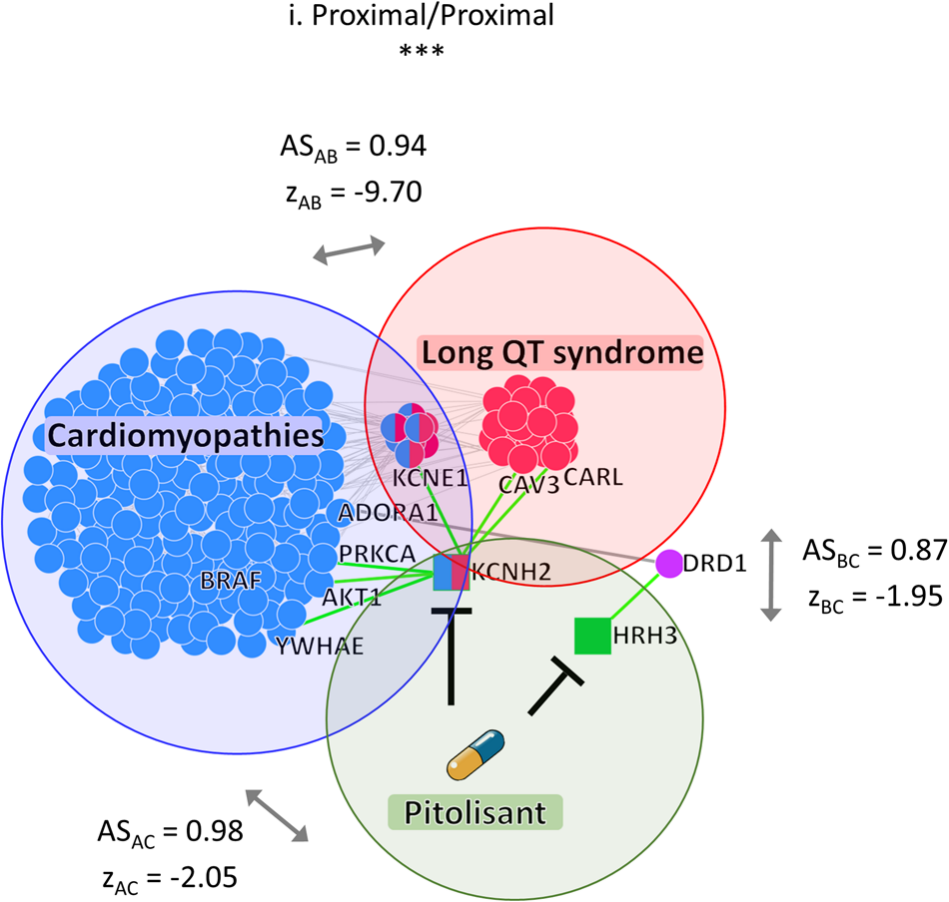
Interaction network of drug targets inducing severe adverse side-effects. Inferred mechanism-of-action network for a selected triplet: cardiomyopathies (disease A), long QT syndrome (side-effect B), and pitolisant (drug C). The *z*_AC,_ and *z*_BC_ denote the *z*-score normalized values of network proximity between the targets of the drug (C) and the genes associated with the disease (A) and with the side-effect (B), respectively; while *z*_AB_ denotes the *z*-score normalized value of network proximity between the genes associated with the disease (A) and with the side-effect (B). AS_AC_, AS_BC_, and AS_AB_ are the corresponding adjusted similarity values. In the interaction network, red circles refer to side-effect-associated genes, blue circles refer to disease-associated genes, squares refer to drug targets (blue and/or red colored if they are shared with the disease and/or side-effect module), and violet circle refers to a first nearest neighbor (that is not disease or side-effect-associated gene) of the drug targets able to connect the drug module to the disease module in the human interactome. Green edges refer to connections between the drug targets and their first nearest neighbors in the human interactome.

By considering cardiomyopathies as a disease of interest, our pipeline identifies 154 potentially adverse drugs predicted to prolong long QT interval (proximal/proximal mode-of-action) and 404 likely safe ones (139 distal/distal and 265 distal/proximal). Among the potentially adverse drugs, we focused on pitolisant, characterized by a proximal/proximal mode-of-action with respect to the side-effect module and the disease module (Fig. 7). We investigated the network neighborhood of its target proteins by highlighting their interactions with the genes associated with cardiomyopathies, as well as with those associated with the long QT syndrome in order to identify specific network regions that may theoretically be altered by the drug’s actions (Fig. 7).

In particular, pitolisant is a potent histamine H3 receptor antagonist/inverse agonist that is approved for the treatment of narcolepsy in adults. It has also been reported to inhibit KCNH2, showing voltage-gated potassium channel activity involved in ventricular cardiac muscle cell action potential repolarization^30^. From the inferred subnetworks, we also observed that KCNH2 indirectly interacts with BRAF via the AKT serine/threonine kinase 1. Clinical studies have correlated the drug class of BRAF inhibitors with QT prolongation^31,32^. Moreover, in a QT study of healthy volunteers, pitolisant (35.6 mg/day) led to a mean increase of 4.2 ms in the QTc interval^33^.

**Fig. 8:**
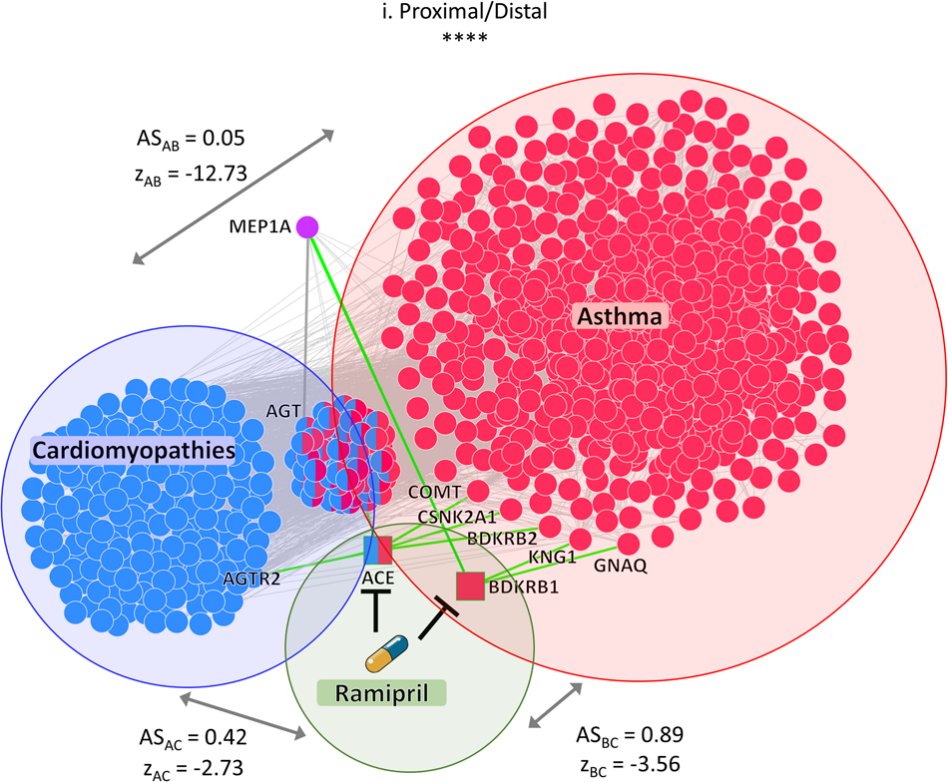
Interaction network of drug targets inducing severe adverse side-effects. Inferred mechanism-of-action network for a selected triplet: cardiomyopathies (disease A), asthma (side-effect B), and ramipril (drug C). The *z*_AC_ and *z*_BC_ denote the *z*-score normalized values of network proximity between the targets of the drug (C) and the genes associated with the disease (A) and with the side-effect (B), respectively; while *z*_AB_ denotes the *z*-score normalized value of network proximity between the genes associated with the disease (A) and with the side-effect (B). AS_AC_, AS_BC_, and AS_AB_ are the corresponding adjusted similarity values. In the interaction network, red circles refer to side-effect-associated genes, blue circles refer to disease-associated genes, squares refer to drug targets (blue and/or red colored if they are shared with the disease and/or side-effect module), and violet circle refers to a first nearest neighbor (that is not disease or side-effect-associated gene) of the drug targets able to connect the drug module to the disease module in the human interactome. Green edges refer to connections between the drug targets and their first nearest neighbors in the human interactome.

Again, considering cardiomyopathies as a disease of interest, our pipeline predicts 286 potentially adverse drugs that can induce asthma (87 with distal/proximal mode-of-action and 199 proximal/proximal mode-of-action) and 272 likely safely repurposable drugs (220 distal/proximal mode-of-action and 52 distal/distal mode-of-action). Among the potentially adverse drugs that our pipeline led us to exclude, we focused on ramipril, characterized by a proximal/distal mode-of-action with respect to the side-effect module and the disease module, respectively (Fig. 8). We investigated the network neighborhood of its target proteins by highlighting their interactions with the genes associated with cardiomyopathies, as well as with those associated with asthma in order to identify specific network regions that may theoretically be altered by the drug’s actions. It is worth noting that whether the disease module is distal from the drug module or from the side-effect module (i.e., showing low adjusted similarity values, Fig. 8) does not exclude the possibility that the disease module shares nodes with the drug or side-effect module, as we observed from the topological network structure of their interconnectedness (Fig. 8).

Yet, the interaction network shows that both of the targets of ramipril (i.e., ACE and BDKRB1) are asthma-associated genes and, thus, the drug module is directly linked to the side-effect module (network-based proximity = 0, AS_BC_ > 0.5 in Fig. 8). By contrast, BDKRB1 is not a cardiomyopathies-associated gene, but reaches the disease module via the MEP1A gene, thus increasing the distance between the drug module and the disease module (network-based proximity = 1, AS_AC_ < 0.5 in Fig. 8).

Ramipril is an ACE inhibitor used for the management of hypertension and the reduction of cardiovascular mortality following myocardial infarction in hemodynamically stable patients with clinical signs of congestive heart failure. Owing to its ability to increase bradykinin levels (by impairing its degradation), it is known to induce urticaria, angioedema, cough, and asthma, as well as anaphylaxis^34^. Indeed, bradykinin is a peptide that promotes inflammation, generated by proteolytic cleavage of its kininogen precursor (KNG1). Nonetheless, bradykinin may also regulate many of the beneficial effects of ACE inhibitors^35^. Activation of the kinin system-bradykinin is important for regulating blood pressure and inflammatory reactions via bradykinin’s ability to cause vasodilation and to increase vascular permeability, respectively^36^.

This mechanism of action of ramipril was recovered in the topological structure of the interactions network, where one target of ramipril (BDKRB1) is directly linked to KNG1 and GNAQ proteins (Fig. 8). Both of these genes/gene products are involved in the inflammatory regulation of the transient receptor potential (TRP) channel, which orchestrates various cellular processes, including cell differentiation, cytokine production, and cytotoxicity. The involvement of the TRP channel in the transition of immune and inflammatory responses from an early defensive response to a chronic pathological condition is also emerging as a putative underlying mechanism in several (cardiovascular and other) diseases^37^.

In summary, we proposed a pipeline to unveil a list of drug candidates for a particular disease (e.g., cardiovascular diseases) that are unlikely to produce specific adverse side-effects (e.g., long QT syndrome). The rationale underlying the study is that drugs whose targets are proximate to the network neighborhood of the side-effect module are more likely to trigger the side-effect, and, thus, a distal relationship between drug targets and side-effect modules is preferred. This assumption has been computationally supported by different criteria, i.e., statistical, biological, as well as topological criteria (Fig. 4 and Supplementary Fig. 4) for two analyzed side-effects (e.g., long QT syndrome and asthma). However, the intrinsic nature of these computational criterion necessarily leads to the presence of false positives that in this context would be those drugs that, while classified as drugs inducing a severe side-effect, actually modulate gene expression or protein function in the side-effect module so as to attenuate the side-effect. Unfortunately, there is currently no way to reduce the number of these false positives. Doing so would require: (i) knowledge about the directional changes in gene expression or protein function caused by drugs known to induce the side-effect in a specific cell line or tissue (gene signature); (ii) knowledge of the effect of the drug on the expression or function of these genes/gene products in the same cell line or tissue (drug signature). If a drug was to revert the side-effect-associated gene signature, it could prevent or mitigate the side-effect (negative correlation between drug signature and disease signature). By contrast, if the drug modulated the side-effect-associated gene expression or protein function in the same direction of the gene signature, it could trigger the side-effect (positive correlation between drug signature and disease signature). Put another way, without knowledge of the (weighted) directionality of the drug’s effect on side-effect module pathways and function, we must consider the possibility that the drug may also attenuate the side-effect (risk). Again, this corollary to the side-effect module proximity hypothesis is analogous to the drug-target-disease module proximity hypothesis: without knowledge of the (weighted) directionality of the drug’s effect on disease module pathways and function, we must consider the possibility that a repurposed drug may also adversely affect disease manifestations. This shortcoming is a reflection of the lack of adequate a priori information on the (net) directional effects of a drug on disease- or side-effect-module function, which can only be ascertained by in vitro experimentation owing to the incomplete nature of available databases (e.g., CMap^38^ and GEO^39^) in terms of the cell types and signaling pathways that have been reported.

In conclusion, although future work is needed to explore the generalizability of our findings to other diseases, our pipeline offers a powerful, network-based strategy for rational drug repurposing and pave the way for in-silico prediction of side effects of a drug by knowing its targets on the interactome.

## Methods

### Data retrieval

#### Human interactome

The human protein–protein interactome was downloaded from^21^, where the authors assembled their in-house systematic human protein–protein interactome with 15 commonly used databases supported by several types of experimental evidence (e.g., binary PPIs from three-dimensional protein structures; literature-curated PPIs identified by affinity purification followed by mass spectrometry; Y2H, and/or literature-derived low-throughput experiments such as BioGRID^40^, HPRD^41^, MINT^42^, IntAct^43^, InnateDB^44^, signaling networks from literature-derived low-throughput experiments; and kinase-substrate interactions from literature-derived low-throughput and high-throughput experiments). This version of the interactome comprises 217,160 protein–protein interactions connecting 15,970 unique proteins.

#### Disease–gene associations

Disease-associated genes were downloaded from Phenopedia^18^, which collects gene associations for 3255 diseases (last version 6.5.1 released on April 27, 2020). For some diseases of interest for which no associated genes were found in Phenopedia, we integrated disease–gene associations available from DisGeNet^19^, which includes 1,134,942 gene-disease associations between 21,671 genes and 30,170 diseases and traits (last version 7.0 released on June 2020). Genes associated with long QT syndrome were obtained from ref. ^27^. A total of 5476 genes associated with 11 diseases/side effects (i.e., arrhythmia, diabetes mellitus, cardiomyopathies, heart arrest, coronary artery disease, coronary heart disease, angina pectoris, myocardial infarction, cerebral arterial diseases, long QT syndrome, drug-induced asthma) were obtained (Table 2).

**Table 2:** List of the analyzed diseases along with the number of disease-associated genes.

Disease	Number of disease genes
Diabetes mellitus	4205
Asthma	1315
Coronary artery disease	1251
Myocardial infarction	930
Arrhythmia	383
Cardiomyopathies	335
Cerebral arterial diseases	304
Angina pectoris	167
Cardiac arrest	147
Long QT syndrome	47
Coronary heart disease	24

#### Drug–target interactions

Drug–target interactions were acquired from DrugBank^20^, which contains 13,563 drug entries, including 2627 approved small molecule drugs, 1373 approved biologics, 131 nutraceuticals, and over 6370 experimental drugs (version 5.1.6 released on April 22, 2020). The targets’ Uniprot IDs provided by DrugBank were mapped to Entrez gene IDs by using the Ensembl BioMart tool (https://www.ensembl.org/), yielding a total of 2165 genes interacting with 1873 drugs.

#### Drug medical indications

The original approved medical indications for the drugs were acquired from the Therapeutic Target Database (TTD)^45^ (last version released on June 1, 2020), which includes information about 5059 drugs associated with 1136 disease classes.

#### Drug-induced side-effects

The known drug-induced side-effects were acquired from SIDER (last version 4.1 released on October 21, 2015)^26^, which includes aggregated information from public resources and package inserts of 5868 side-effects for 1430 drugs. Only drug-side-effect pairs associated with preferred terms (PT) according to the MedDRA dictionary^46^ were retained, yielding a total of 141,100 associations of 4251 side-effects with 1344 drugs. In addition, drugs known to induce long QT syndrome were also acquired from^27,28^ and integrated with the information provided by SIDER, as were drugs known to induce asthma.

### SAveRUNNER algorithm

Recently, we developed a novel network-based algorithm for drug repurposing called SAveRUNNER (Searching off-lAbel dRUg aNd NEtwoRk)^16,17^, with the aim of screening efficiently novel potential indications for currently marketed drugs against diseases of interest, and optimizing the efficacy of putative validation experiments. Taking as input the human interactome network, the list of disease–gene associations, and the list of drug–target interactions, SAveRUNNER predicts drug-disease associations by quantifying the interplay between the drug targets and disease-associated proteins in the human interactome via a novel network-based similarity measure (denoted adjusted similarity) defined as follows:

1 $${\mathrm{AS}}(p) = \frac{1}{{1 + e^{ - c\left[ {\frac{{(1 + {\mathrm{QC}})(m - p)}}{m} - d} \right]}}}$$

where *p* is the network proximity measure defined in^21^:

2 $$p\left( {T,S} \right) = \frac{1}{{\left\| T \right\|}}\mathop {\sum}\nolimits_{t{\it{\epsilon }}T} {\mathop {{\min }}\limits_{s{\it{\epsilon }}S} d(t,s)}$$

*p*(*T,S*) represents the average shortest path length between drug targets *t* in the drug module *T* and the nearest disease genes *s* in the disease module *S*; QC is a quality cluster score that rewards associations between drugs and diseases located in the same network cluster; *m* is max(*p*); *c* and *d* are the steepness and the midpoint of AS(*P*), respectively. A comprehensive description of SAveRUNNER methodology can be found in^16,17^.

### Identification of network modules

In order to test whether the analyzed diseases form a statistically significant disease module for each analyzed disease or side-effect, the lists of disease/side-effect-associated genes were mapped onto the human interactome, the corresponding subnetwork was extracted, and the following three metrics were computed: (1) the size of the largest connected component (LCC); (2) the number of interactions in the LCC; and (3) the total number of interactions (edges). In order to complement these metrics with a measure of statistical significance, we evaluated the probability that a given list of disease/side-effect-associated genes was localized within a certain network neighborhood greater than expected by chance^25^. Specifically, we randomly selected groups of proteins in the human interactome network with the same size and degree distribution as the original list of disease/side-effect-associated genes and we computed the three above-mentioned metrics. This procedure was repeated 1000 times, and we derived three distributions for all three metrics corresponding to the subgraph induced by the random gene set. The three metrics calculated for the original list of disease genes were *z*-score-normalized with respect to the corresponding reference random distribution. Subsequently, the *p*-value for the given *z* statistic was calculated, expecting a *p*-value ≤ 0.05 for genes forming a statistically significant disease module^25^. We found that all of the analyzed sets of disease genes form statistically significant modules (i.e., all three metrics were statistically significant) in the human interactome (Table 1).

### Statistical analysis

In order to test if the drugs predicted by SAveRUNNER to be associated with a side-effect were enriched in drugs that are known to produce it, we performed a hypergeometric test computing the following statistic (Fig. 4B):

3 $$p = \mathop {\sum}\nolimits_{i = X}^S {\frac{{\left( {\begin{array}{*{20}{c}} K \\ i \end{array}} \right)\left( {\begin{array}{*{20}{c}} {U - K} \\ {S - i} \end{array}} \right)}}{{\left( {\begin{array}{*{20}{c}} U \\ S \end{array}} \right)}}}$$

where *U* is the universe dimension, i.e., the number of drugs (1344) retrieved from SIDER; *K* is the property, i.e., the number of drugs previously reported to induce the side-effect of interest; *S* is the selection, or the number of drugs predicted by SAveRUNNER for the side-effect of interest with adjusted similarity ≥0.5; and *X* is the intersection, that is the number of drugs predicted by SAveRUNNER for the side-effect of interest with adjusted similarity ≥0.5 intersecting with those that are known to induce it.

### ROC curve analysis

The performance of our pipeline in predicting drugs known to induce a side-effect as well as known drugs–disease associations was evaluated in terms of the receiver operating characteristic (ROC) curve analysis. In both cases, the predictions were ranked according to decreasing adjusted similarity values, and a truth table was built by considering the “real association” according to SIDER^26^ or TTD database information^45^:1 if the prediction (i.e., drug-induced side-effect or drug-disease association) is known, 0 otherwise. For a specified adjusted similarity threshold, the true-positive rate (i.e., sensitivity) was computed as the fraction of known associations that are correctly predicted, while the false-positive rate (i.e., 1-specificity) was computed as the fraction of unknown associations that are predicted. The ROC probability curve was defined based on these measures at different thresholds and the corresponding area under the curve (AUC) was computed. The greater the AUC, the better the algorithm is at distinguishing between the two analyzed classes (i.e., drugs known to induce the side-effect *versus* drugs unknown to induce the side-effect or known drug-disease associations *versus* unknown drug-disease associations).

## Supplementary information

SupplementaryInformation

Supplementary Table 1

Supplementary Table 2

Supplementary Table 3

Supplementary Table 4

Supplementary Table 5

Supplementary Table 6

## Data Availability

All data generated or analyzed during this study are included in the manuscript and its Supplementary files.

## Code availability

SAveRUNNER code is open-source and it is available at https://github.com/sportingCode/SAveRUNNER.git.
