# Supplementary material for: Comprehensive network medicine-based drug repositioning via integration of therapeutic efficacy and side effects
Source: NPJ Syst Biol Appl. 2022 Apr 20;8:12. doi: 10.1038/s41540-022-00221-0 (PMC9021283; doi:10.1038/s41540-022-00221-0)
Supplement: Supplementary file 1 — SupplementaryInformation [file 41540_2022_221_MOESM1_ESM.pdf]

Comprehensive Network Medicine-based Drug Repositioning via Integration of Therapeutic Efficacy and Side Effects

Paola Paci, Giulia Fiscon, Federica Conte, Rui-Sheng Wang Diane E. Handy, Lorenzo Farina, Joseph Loscalzo

Supplementary Information

This file lists the description of the Supplementary Tables provided as supplementary material (.xlsx file), as well as all the Supplementary Figures.

Supplementary Tables

Supplementary Tables 1: Drug-disease network. This table reports the drug-disease network obtained by running SAveRUNNER.

Supplementary Table 2: Removed drugs that are potentially adverse. This table reports the list of drugs that have been removed from the potentially repurposable drugs list predicted by SAveRUNNER because of their inducing long QT syndrome (first sheet) or asthma (second sheet), along with any prior evidence that these drugs are known to induce the side-effect from SIDER or from literature studies (with known SE), or computational evidence predicting it to induce the side-effect by our pipeline (with predicted SE). For each drug, a list of additional induced side-effects is also shown.

Supplementary Table 3: Repurposable drugs unlikely to prolong the QT interval. This table reports the final list of the repurposable drugs for each analyzed disease after the removal of potentially adverse drugs inducing long QT syndrome. The original medical indications obtained from the TTD database, as well as the drug-induced side-effects retrieved from the SIDER database or from the literature, are also reported.

Supplementary Table 4: Repurposable drugs unlikely to induce asthma. This table reports the final list of the repurposable drugs for each analyzed disease after the removal of potentially adverse drugs inducing asthma. The original medical indications obtained from the TTD database, as well as the drug-induced side-effects retrieved from the SIDER database or from the literature, are also reported.

Supplementary Table 5: Drug's mode of action for cardiomyopathies. This table reports the list of drugs predicted to be repurposable for cardiomyopathies along with their adjusted similarity values. The table also reports the mode of drug action with respect to the long QT syndrome module and asthma (i.e., proximal/distal, proximal/proximal, distal/proximal, or distal/distal) (cf. Figure 5).

Supplementary Table 6: Drug's mode of action for arrythmia. This table reports the list of drugs predicted to be repurposable for arrythmia along with their adjusted similarity values. The table also reports the mode of drug action with respect to the long QT syndrome module and asthma module (i.e., proximal/distal, proximal/proximal, distal/proximal, or distal/distal) (cf. Supplementary Figure 5).

Supplementary Figures

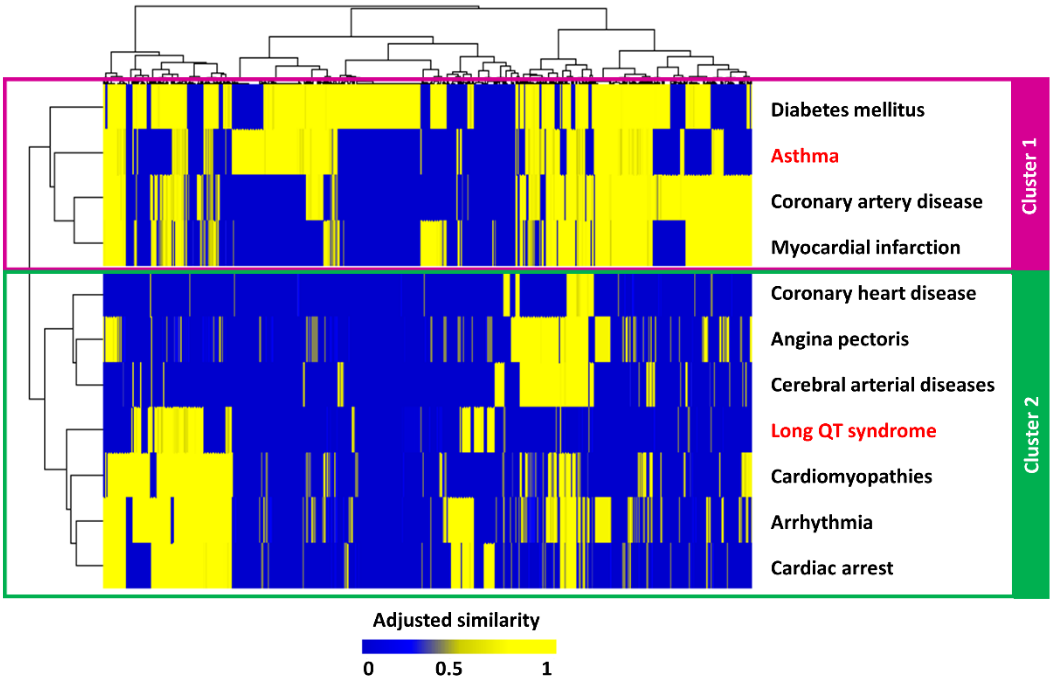

Supplementary Figure 1. Dendrogram and heatmap of the drug-disease network. The drug-disease network composed of 1,552 nodes (i.e., 11 diseases and 1,541 drugs) connected by 6,436 links is naturally rendered as a matrix where the columns correspond to the drugs predicted by SAveRUNNER to be repurposable for the diseases analyzed in this study, and the rows correspond to the diseases. The heatmap color key denotes the adjusted similarity between drug targets and disease genes in the human interactome, increasing from blue (less similar) to yellow (more similar). The adjusted similarity values are clustered according to rows (diseases) and columns (drugs) by a complete linkage hierarchical clustering algorithm and by using the Euclidean distance as distance metric.

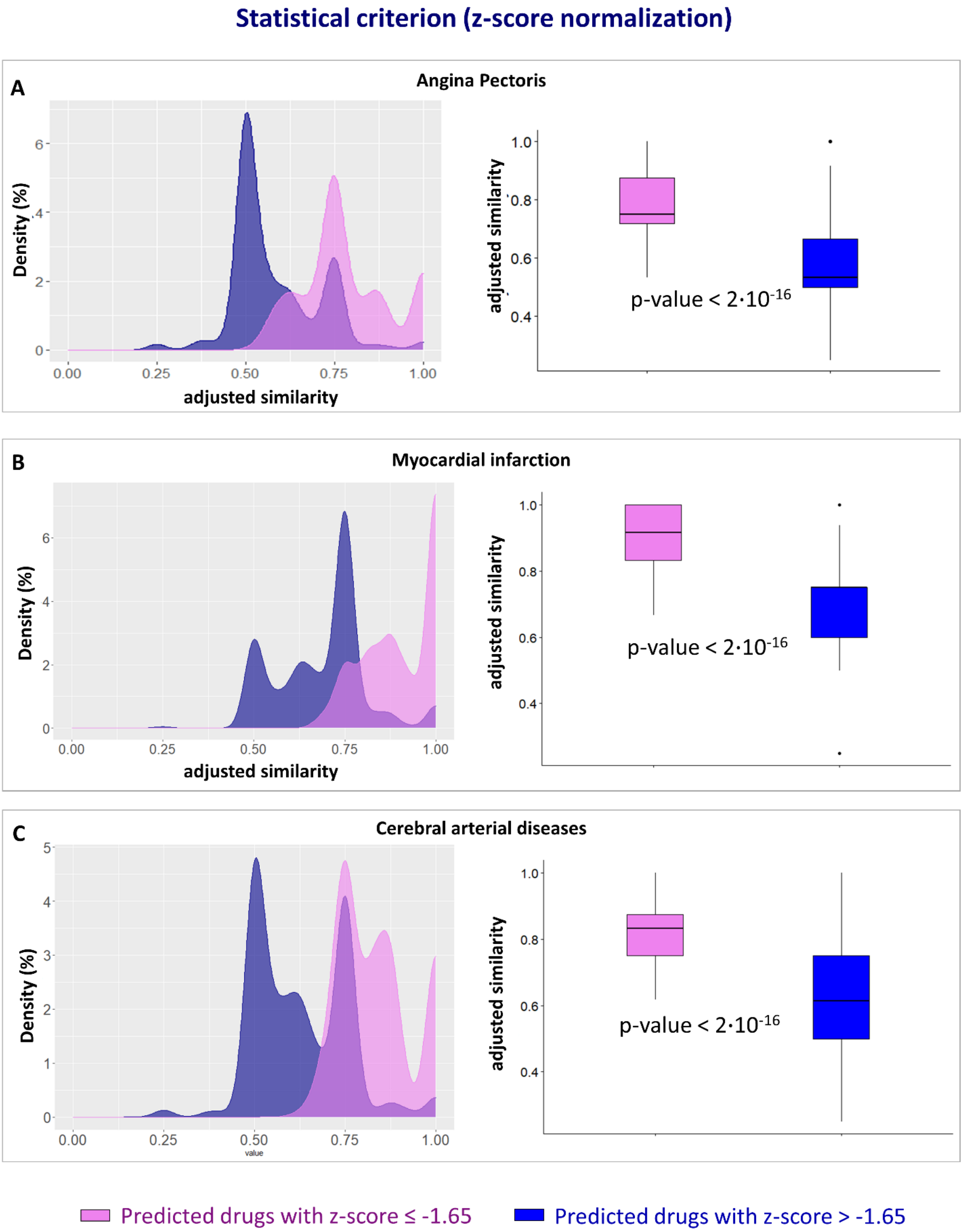

Supplementary Figure 2. Drug-disease proximity criterion. Kernel density estimate plots (left) and box plots (right) for the adjusted similarity values of the statistically significant drugs (pink plot, corresponding to a z-score ≤-1.65) and non-statistically significant drugs (blue plot, corresponding to a z-score > -1.65) predicted by SAveRUNNER as repurposable for angina pectoris (A), myocardial infarction (B), and cerebral arterial diseases (C). For each disease, a t-test was used to compare the two distributions and statistical significance was found in each case (p-value < 0.05). Box-plot elements are so defined: center line, median; box limits, upper and lower quartiles; points, outliers.

A B

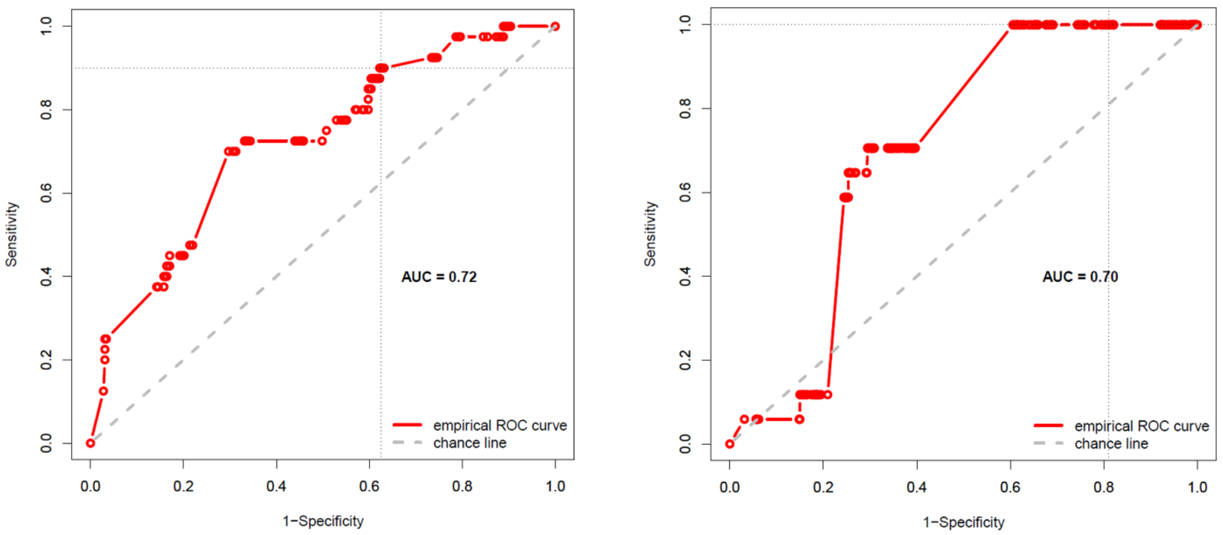

Supplementary Figure 3. ROC curve analysis for identifying known drug-side-effect associations (A) and known drug-disease associations (B). The plots report the true positive rate (i.e., sensitivity) vs. the false positive rate (i.e., 1-specificity) at various threshold settings. The dashed grey line represents the line of no-discrimination between two classes: **(A)** drugs known to induce the side-effect *versus* drugs not known to induce the side-effect; **(B)** drugs known to be associated with the disease *versus* drugs not known to be associated with the disease. The intersection between the black dotted lines corresponds to an adjusted similarity equal to 0.5.

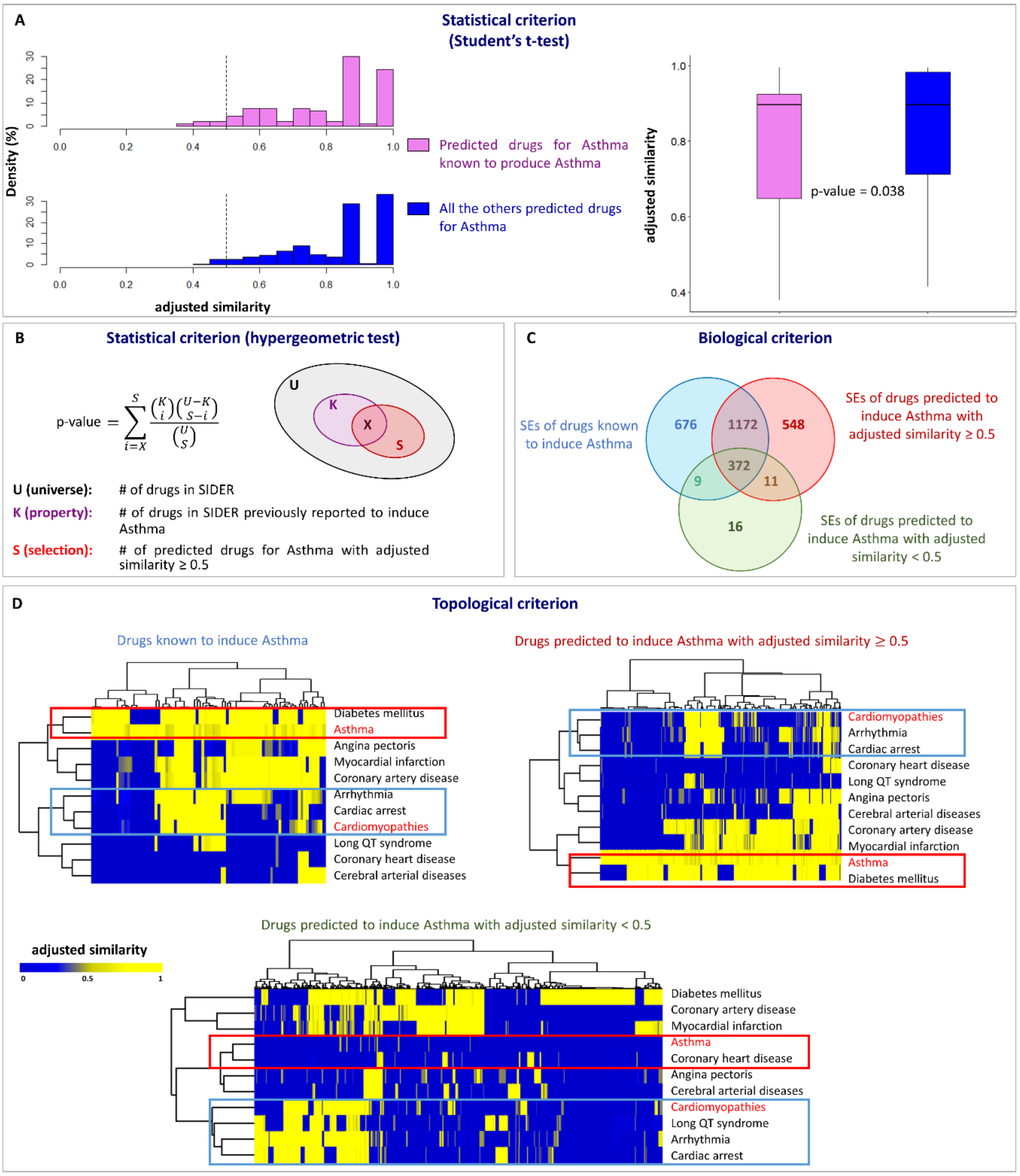

Supplementary Figure 4. Drug-side-effect proximity criterion. A) Statistical criterion (Student's t-test): histogram (left) and box plot (right) of the adjusted similarity values of the drugs predicted by SAveRUNNER that are known to induce asthma (pink bar), and for all the other predicted drugs (blue bar). T-test was used to compare the two distributions. Box-plot elements are so defined: center line, median; box limits, upper and lower quartiles; points, outliers. **B)** Statistical criterion (hypergeometric test): illustration of the ensembles considered for the hypergeometric test calculation. **C)** Biological criterion: Venn diagram among the side-effects (SEs) of the drugs known to induce asthma (blue ensemble), of the drugs predicted by SAveRUNNER to induce asthma with adjusted similarity ≥ 0.5 (red ensemble) and with adjusted similarity < 0.5 (green ensemble). **D)** Topological criterion: dendrogram and heatmap of the drug-disease network predicted by SAveRUNNER including drugs known to induce asthma (top left), drugs predicted to induce asthma with adjusted similarity ≥ 0.5 (top right), and drugs predicted to induce asthma with adjusted similarity < 0.5 (bottom).

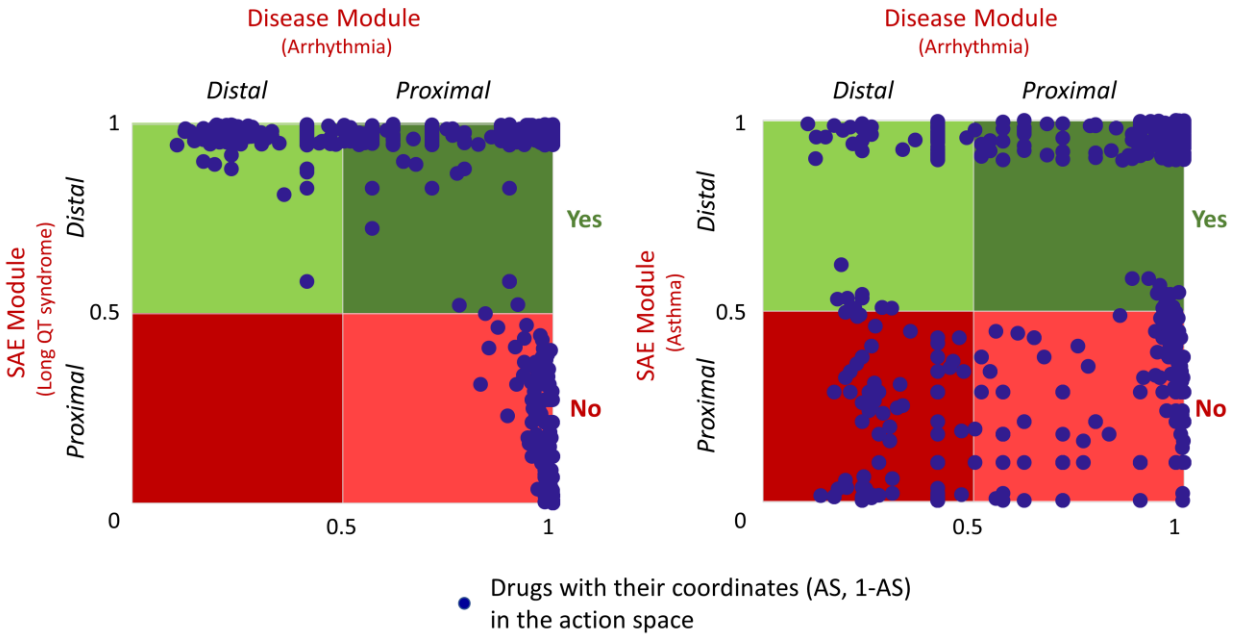

Supplementary Figure 5. Drug-action map for Arrhythmia. Scatter plot of AS for the disease module on the x-axis, and (1-AS) for SAE module (i.e., [left] long QT syndrome, [right] drug-induced asthma) on the y-axis. The four possible modes of action are colored with shades of red and green according to their predicted adverse or beneficial effects, respectively. Abbreviations: AS=adjusted similarity, and SAE=serious adverse effect.
